# Antitumour Metallocenes: Effect of DMSO on the Stability of Cp_2_TiX_2_ and Implications for Anticancer Activity

**DOI:** 10.1155/MBD.1998.207

**Published:** 1998

**Authors:** George Mokdsi, Margaret M. Harding

**Affiliations:** School of Chemistry University of Sydney N.S.W. 2006 Australia

## Abstract

The rate of hydrolysis of the aromatic rings of Cp_2_TiX_2_ [X = CI **1**, O_2_CCCl_3_ **8** and O_2_CCH_2_NH_3_Cl **13**], in aqueous solutions, 10%DMSO and 100% DMSO have been studied by ^1^H NMR spectroscopy. Rapid hydrolysis of both the carboxylate and cyclopentadienyl ligands in Cp_2_TiX_2_[X = O_2_CCCl_3_,O_2_CCH_2_NH_3_Cl] occurs in DMSO to give biologically inactive species. The rate of these reactions are concentration dependent as dilution of these samples with saline or water to give the therapeutic conditions of 10%DMSO/90%H_2_O slows the hydrolysis chemistry. In contrast, samples of Cp_2_TiX_2_ [X = CI **1**, O_2_CCH_2_NH_3_Cl**13** ], dissolved in water give solutions containing the presumed antitumour active species in which the halide or glycine ligands have been hydrolysed but the Cp rings remain metal bound.


### ANTITUMOUR METALLOCENES: EFFECT OF DMSO ON THE STABILITY OF $Cp_2TiX_2$ AND IMPLICATIONS FOR ANTICANCER ACTIVITY

George Mokdsi and Margaret M. Harding*

School of Chemistry, University of Sydney, N.S.W. 2006, Australia

**Abstract**

The rate of hydrolysis of the aromatic rings of $Cp_2TiX_2$ [X = Cl **1**, $O_2CCCl_3$ **8** and $O_2CCH_2NH_3Cl$ **13**], in aqueous solutions, 10%DMSO and 100% DMSO have been studied by $^1H$ NMR spectroscopy. Rapid hydrolysis of both the carboxylate and cyclopentadienyl ligands in $Cp_2TiX_2$ [X = $O_2CCCl_3$, $O_2CCH_2NH_3Cl$] occurs in DMSO to give biologically inactive species. The rate of these reactions are concentration dependent as dilution of these samples with saline or water to give the therapeutic conditions of 10%DMSO/90%$H_2O$ slows the hydrolysis chemistry. In contrast, samples of $Cp_2TiX_2$ [X = Cl **1**, $O_2CCH_2NH_3Cl$ **13**], dissolved in water give solutions containing the presumed antitumour active species in which the halide or glycine ligands have been hydrolysed but the Cp rings remain metal bound.

### Introduction

The metallocene dihalides $Cp_2MX_2$ (Cp =$\eta^5$-$C_5H_5$; M = Ti, Mo, Nb, V; X = F, Cl, Br, I, NCS, $N_3$, Y) are a relatively new class of small, hydrophobic organometallic anticancer agents that exhibit antitumour properties against numerous cell lines including leukemia's P388 and L1210, colon 38 and Lewis lung carcinomas, B16 melanoma, solid and fluid Ehrlich ascites tumours and several human colon and lung carcinomas transplanted into athymic mice [1,2,3]. Titanocene dichloride ($Cp_2TiCl_2$) **1** has been the most widely studied metallocene and the drug entered phase I clinical trials in late 1991 [4]. Both biological and chemical studies support interaction with DNA being involved in the mechanism of antitumour action. However, the active species responsible for antitumour activity *in vivo* has not been identified.

Structure-activity studies, in which both the chloride ligands (Tables 1, 2) and the Cp rings have been modified, are consistent with the formation of a hydrolysed species ("$Cp_2Ti^{2+}$") [5] as the active species *in vivo* which is assumed to complex to DNA and interfere with normal cell replication processes. $Cp_2TiCl_2$ **1** undergoes rapid halide and cyclopentadienyl (Cp) hydrolysis to form cyclopentadiene (CpH) and dicyclopentadiene, particularly at pH >4.0 [6]. However, these hydrolysis products cause non-specific, local tumour inhibiting effects and do not exhibit the systemic antitumour activity of $Cp_2TiCl_2$ [1,7]. The partially hydrolysed derivative $CpTiCl_3$, which lacks one of the Cp rings exhibits reduced activity [8], while substitution of the Cp rings, or bridging of the two Cp rings resulted in significant loss of antitumour activity [1,9]. Thus, the unsubstituted Cp rings are an important structural feature required to maintain antitumour activity.

In contrast, modification of the halide ligands is possible, provided the Ti-X bond remains labile in aqueous solution. Thus derivatives $Cp_2TiX_2$ (Table 1) retain antitumour activity, with reduced or no activity in several cases (eg. X = *p*-nitrophenoxy **10**) attributed to pseudohalide ligands that are too strongly bound to the titanium centre thus preventing dissociation in aqueous systems [10]. Introduction of hydrophilic and charged X ligands (e.g. **7**,**8** and **9**; Table 1) has provided a mechanism whereby the aqueous solubility of complexes $Cp_2TiX_2$ may be improved, without significant perturbation of antitumour properties [11]. Very recently the synthesis and biological activity of the highly water soluble amino acid derivatives **13** and **14** was reported [12,13]. While in the solid state, complex **13** crystallises with the carboxylate groups coordinated to the metal centre, in solution we have shown that the glycine ligands are only weakly coordinated to the metal centre, and that, as in the case of $Cp_2TiCl_2$ (aq), the predominant species present in aqueous solutions of **13** at pH 2-5 is "$Cp_2Ti^{2+}$" [14]. However, in contrast to all previously studied titanocenes containing weakly coordinating halide or pseudo halide ligands, antitumour testing of this derivative against Ehrlich ascites tumours did not show the expected 100% cure rates and activity levels of only 50% were achieved (Table 1).

In an effort to further define the key structural elements required in $Cp_2TiX_2$ to retain antitumour activity, this paper reports a study of the hydrolysis chemistry [15] of titanocene derivatives $Cp_2TiX_y$ (X = Cl **1**, $O_2CCCl_3$ **8** and $O_2CCH_2NH_3Cl$ **13**) in water and aqueous DMSO mixtures. Particular attention was paid to the therapeutic conditions of 10% DMSO. The results show that the effect of solvent is important, as in the case of the carboxylate ligands, Cp hydrolysis is promoted in DMSO, leading to reduced amounts of the putative active species "$Cp_2Ti^{2+}$" in solution. The results provide further molecular level information that is essential for the design of related metallocene based anticancer drugs.

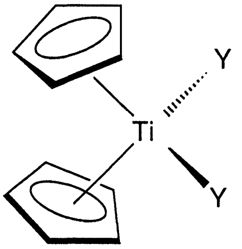

**1** Y = Cl

**2** Y = O-C(=O)-$CH_2NH_3Cl$

**3** Y = O-C(=O)-$CCl_3$

### MATERIALS AND METHODS

### *General*

Titanocene dichloride **1** was obtained from the Aldrich chemical company. The bisglycine analogue **13** was prepared from titanocene dichloride **1** according to the literature procedure [12], and purified and characterised as reported in [14]. Derivative **8** was prepared according to the literature procedure [11] and was purified by recrystallisation in 87% yield; m.p. 173-174 $^0$C. $^1$H NMR 200 MHz ($CDCl_3$) δ 6.67 (s, 10H, Cp). IR spectrum (KBr disc, $cm^{-1}$): 3114m; 1688s (C=O); 1444m; 1393m; 1333s; 1303s; 1020m; 961m; 869m; 839m; 732s; 680s. EI MS m/z 437 ($CpTi(OOCCCl_3)_2^+$, 4%); 341 and 339, ($(Cp_2Ti(OOCCCl_3)^+$, 63); 311, ($CpTi(OOCCCl_3)Cl^+$, 52); 248, ($Cp_2TiCl_2^+$, 11); 213 ($Cp_2TiCl^+$, 30); 183, ($CpTiCl_2^+$, 100); 178, ($Cp_2Ti^+$, 3); 148, ($CpTiCl^+$, 2). $^1$H NMR spectra were recorded on a Bruker AC200 NMR spectrometer and were referenced to TSP (0.00 ppm). DMSO refers to $d_6$-DMSO.

**Table 1** : Effect of X ligand on biological activity of $Cp_2TiX_2$ against fluid Ehrlich ascites tumour; samples administered in 10%DMSO/saline

| Compound | X ligand | Optimum Cure Rate (%) | Optimum dose range (mg/kg) | $LD_{50}$ (mg/kg) | Ref. |
|---|---|---|---|---|---|
| **1** | Cl | 100 | 40-60 | 100 | 1,19 |
| **2** | F | 100 | 40 | 60 | 1,19 |
| **3** | Br | 100 | 40-80 | 135 | 1,19 |
| **4** | I | 100 | 60-100 | 145 | 1,19 |
| **5** | NCS | 100 | 80 | 135 | 1,19 |
| **6** | $N_3$ | 100 | 50-70 | 95 | 1,2 |
| **7** | $SC_6H_4NH_3Cl$ | 100 | 60-140 | 175 | 2,11 |
| **8** | $OCOCl_3$ | 100 | 100-360 | 440 | 1,11 |
| **9** | cis $O_2CCH{=}CHCO_2H$ | 100 | 60-120 | 170 | 1,11 |
| **10** | *p*-$NO_2C_6H_4O$ | 10 | 180-240 | 260 | 1 |
| **11** | $OC_6F_5$ | 100 | 340-360 | 480 | 1,10 |
| **12** | $SC_6F_5$ | 100 | 120-180 | 260 | 1,10 |
| **13** | $O_2CCH_2NH_3Cl$ | 50 | 100-120 | >160 | 12,13 |
| **14** | (L)- $O_2CCH(CH_3)NH_3Cl$ | 50 | 160 | 185 | 12,13 |

### Hydrolysis Experiments

The general procedure involved dissolving 5- 15 μmol of the complex in 500 μl $D_2O$ or DMSO, or 10%DMSO/90%$D_2O$. For samples in 10%DMSO, the solid was first dissolved in DMSO, and then diluted with $D_2O$ to the required volume. Sonication was carried out with an Elma Transsonic Digital Bath. The solution pD was adjusted with DCl and NaOD. pD values were measured using a Beckman F11 meter and a Mettler NMR tube pH probe and are related to the pH meter reading by the formula pD = pH(meter reading) + 0.4 [16]. Measured pD values are ± 0.3 due to fluctuations in sample pH which occurred over 30 min. $^1H$ NMR spectra were recorded at time intervals with any developing precipitate ignored. The rate of Cp hydrolysis displacement was estimated by integration of one of the two multiplets arising from free cyclopentadiene $C_5H_5D$ (6.5 and 6.6 ppm at pD = 6.2; 6.45 and 6.55 ppm in DMSO) versus the metal-bound $C_5H_5$ signals (typically at ~6.65 ppm). In the case of **13** an estimate of the % hydrolysis was also made from integration of the unbound glycine peak versus the metal bound-Cp signal as described in the results section. Attempts to use TSP as an internal reference were unsatisfactory as coordination of TSP to the metallocene was observed.

**Table 2** : Effect of X ligand on biological activity of $Cp_2TiClX$ against fluid Ehrlich ascites tumour; samples administered in 10%DMSO/saline

| Compound | X ligand | Optimum Cure Rate (%) | Optimum dose range (mg/kg) | $LD_{50}$ (mg/kg) | Ref. |
|---|---|---|---|---|---|
| **15** | 2,3,4-trichlorophenoxy | 50 | 60-140 | 200 | 1,10 |
| **16** | *o*-$SC_6H_4CH_3$ | 25 | 30-60 | 60 | 1,8 |
| **17** | *o*-$SC_6H_4NH_2$ | 13 | 50-100 | 100 | 1,8 |

### RESULTS

The rate of Cp hydrolysis of both **1** and **13** in water at different pD values has been reported previously (see Table 3 [6,14]). Preliminary data for the hydrolysis of a number of carboxylate complexes, including **8** have been reported. However, no details of the aqueous solubility of **8**, which is reported to be poor in an independent study [11], were given, and NMR spectroscopy was not used to characterise the proposed complexes. There are no systematic studies of the hydrolysis of **1**, **8** or **13** in aqueous DMSO or neat DMSO.

The rate of Cp hydrolysis in **1, 8, 13** was measured using $^1H$ NMR spectroscopy at regular time intervals in $D_2O$, DMSO and 10%DMSO/$D_2O$ at 25 $^0C$, using similar methods to those previously reported [14, 17]. Thus, an estimate of the Cp hydrolysis may be calculated from the relative intensities of the new multiplets due to cyclopentadiene and dicyclopentadiene in solution. In addition, hydrolysis is also apparent by formation of precipitates with time. As in previous studies [ref], full characterisation of these precipitates has not been possible due to their poor aqueous solubility, but formation of oligomers and bridged species containing Cp rings cannot be ruled out. Hence the amount of precipitation was also used as an indicator of the amount of hydrolysis occurring in solution. In the case of **1** and **8** the amount of precipitate could only be estimated visually or by isolation and weighing the precipitate. However, in the case of **13**, a more accurate estimate was possible by integration of the glycine $CH_2$ peak versus the cyclopentadienyl protons. Fully dissolved complex **13** gives by integration a ratio of 10 : 4 = Cp : $CH_2$. As precipitation occurred the Cp signal decreased relative to the glycine peak consistent with formation of degradation products derived from the Cp rings.

### Hydrolysis in Water

The rate of Cp hydrolysis of complexes **1** and **13**, including mixed experiments containing equal amounts of both complexes, have been reported previously [14], and is summarised in Table 3. While the hydrolysis of both **1** and **13** is accompanied by formation of a minor amount of insoluble precipitate at ~ pD 2, all of the dissolved material contains the Cp rings bound to the metal. Attempts to carry out similar experiments with **8** confirmed previous reports of poor aqueous solubility the complex [11]. While the solubility was not quantified, the trichloroacetate derivative **8** appeared to be less soluble than titanocene dichloride **1**.

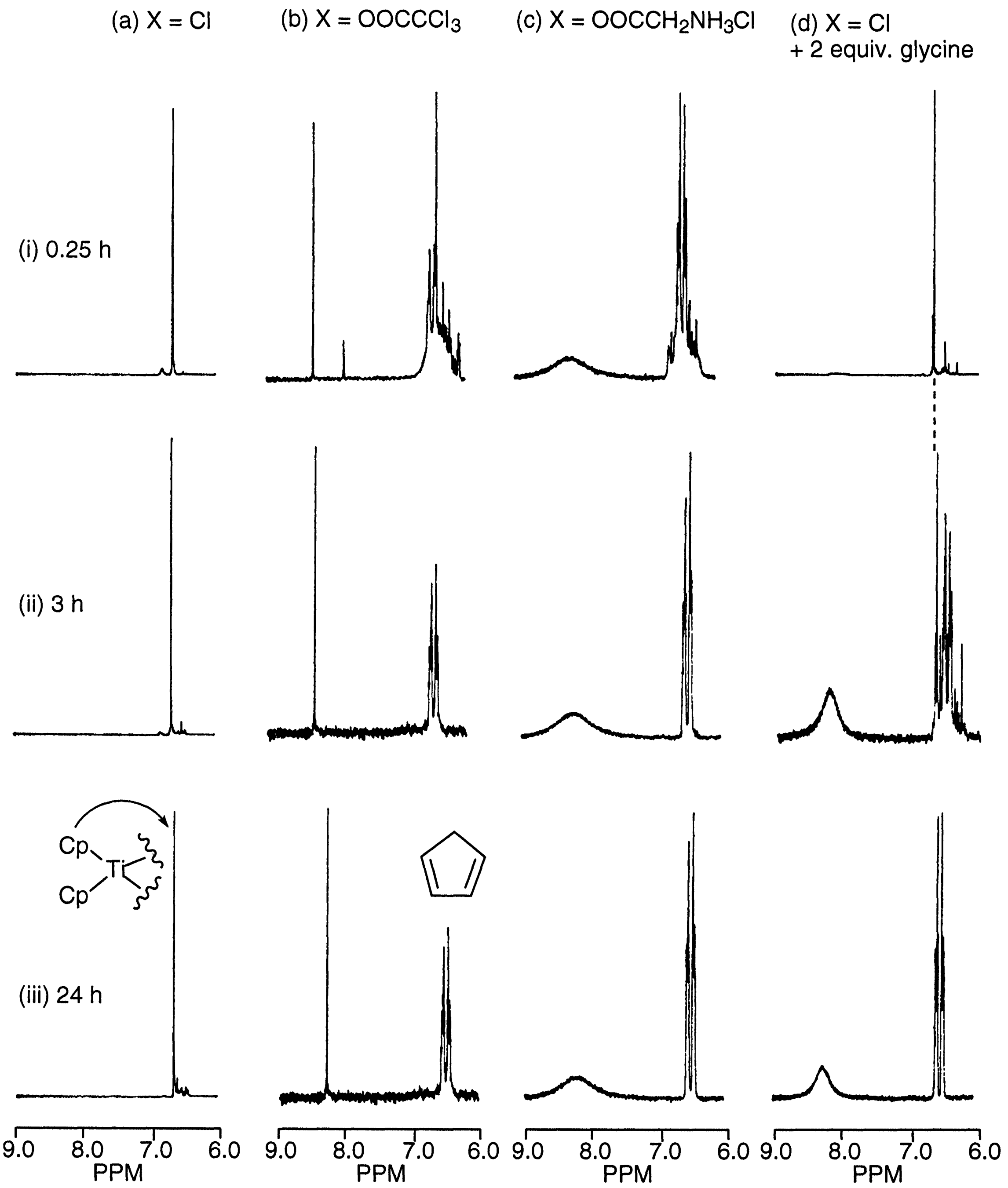

**Figure 1:** Aromatic region of $^1H$ NMR spectra (200 MHz, $d_6$-DMSO, 25 °C) of $Cp_2TiX_2$ complexes (a) **1**, (b) **8** (c) **13** (d) **1** with 2 equivalents of glycine. Spectra recorded at (i) t = 0.25 h (ii) 3 h and (iii) 24 h after complexes dissolved. Vertical axis is not to scale.

### *Hydrolysis in 100% DMSO*

Table 3 summarises the results obtained for the complexes $Cp_2TiX_2$ (X = Cl **1**, $O_2CCCl_2$ **8**, glycine **13**; 10 μmol) in 100%DMSO. All complexes readily dissolved in this solvent with minimal precipitation observed over a 24 h period. In each case, there are 2 competing hydrolysis pathways that need to be considered (i) halide hydrolysis and (ii) ring (Cp) hydrolysis.

In the case of titanocene dichloride **1**, negligible Cp hydrolysis occurred after 15 min and a sharp singlet for the aromatic protons was observed (Figure 1a). After 24 h, by integration of the newly formed multiplets from cyclopentadiene, it was estimated that approximately 30% of the Cp rings in the complex had hydrolysed (Figure 1a). While it is difficult to monitor the rate of halide hydrolysis in these experiments, a new minor downfield DMSO multiplet appeared after 3 h (2.95 ppm) and was assigned to DMSO coordinated to Ti through substitution of one (or both) chloride ligands. This new multiplet increased in intensity at approximately the same rate as the new CpH multiplets.

**Table 3** : Effect of solvent on rates of hydrolysis of aromatic rings in $Cp_2TiX_2$.

| Solvent | DMSO | | $10\%DMSO/D_2O$ | | $D_2O$ (pD ~2-3) [14] | |
|---|---|---|---|---|---|---|
| X | Reaction Time (h) [a] | % Cp Hydrolysis [15] | Reaction Time (h) | % Cp Hydrolysis | Reaction Time (h)[a] | % Cp hydrolysis [15] |
| | 0.25 | <5 | 0.25 | <2 | - | - |
| Cl | 3 | 7 | 3 | <2 | 1 | 0 |
| **1** | 24 | 30 | 24 | <2 | 24 | <2 |
| | 0.25 | >99 | 0.25 | <2 | -[b] | - |
| $O_2CCCl_3$ | 3 | >99 | 3 | <2 | -[b] | - |
| **8** | 24 | >99 | 24 | <2 | -[b] | - |
| | 0.25 | >99 | 0.25 | <2 | - | - |
| glycine | 3 | >99 | 3 | <2 | 1 | 0 |
| **13** | 24 | >99 | 24 | 20 | 24 | <2 |
| Cl | 0.25 | 40 | - | - | - | - |
| **1** | 3 | 80 | - | - | - | - |
| + 2 equiv gly | 25 | >99 | - | - | - | - |

[a] The time taken after complete dissolution was achieved; [b] Insoluble

In the case of complexes **8** and **13**, rapid Cp hydrolysis occurred within 15 min (Figure 1b,1c), and after 24 h the singlets due to the metal bound Cp rings had completely disappeared and only the characteristic cyclopentadiene (CpH) multiplets were observed. Solutions of **8** also darkened after 24 h. As in the case of **1**, a downfield DMSO multiplets appeared in these experiments and were assigned tentatively to DMSO coordinated to titanium. This DMSO-Ti signal increased in intensity with time and was the predominant species present after 24 h. A new resonance also appeared in the spectra at around 8.4 ppm (Figure 1b, 1c and 1d) and was tentatively assigned as a binuclear degradation species similar to those reported previously, (eg., $[Cp_2Ti(X)O(X)TiCp_2]^{2+}$ [18]). This resonance was sharp in solutions of complex **8** and broad in solutions where glycine was present (Figure 1c and 1d).

In order to establish whether glycine, which appears to rapidly dissociate from the metal when **13** is dissolved in DMSO, promotes the Cp hydrolysis reaction, glycine ($HOOCCH_2NH_2$, 2 mol equivalents) was added to a solution of $Cp_2TiCl_2$ **1** and NMR spectra were recorded over 24 h. After 15 min, approx 40% Cp hydrolysis had occurred, while after 3 h significant Cp hydrolysis (80%) was observed (Figure1d, Table 3). Thus, free glycine does promote the Cp hydrolysis reaction, but the rate is slower than in the case of bisglycine derivative **13** in which the glycine ligands are initially coordinated to the metal centre in the zwitterioninc form.

**Table 4**: Hydrolysis of titanocene complexes dissolved in DMSO and left standing for various lengths of time before adding $D_2O$ to give a 10% DMSO/$D_2O$ solution

| Complex | Time in DMSO (min) [a] | Reaction Time (h) [b] | % Cp Hydrolysis [15] |
|---|---|---|---|
| $Cp_2TiCl_2$ **1** | 0.5 | 0.25 | < 2 |
| | | 3 | < 2 |
| | | 24 | < 2 |
| | 3 | 0.25 | < 2 |
| | | 3 | < 2 |
| | | 24 | < 2 |
| | 10 | 0.25 | < 2 |
| | | 3 | < 2 |
| | | 24 | < 2 |
| $Cp_2Ti(gly)_2$ **13** | 0.5 | 0.25 | 10 |
| | | 3 | 10 |
| | | 24 | 10 |
| | 3 | 0.25 | 25 |
| | | 3 | 25 |
| | | 24 | 40 |
| | 10 | 0.25 | 35 |
| | | 3 | 40 |
| | | 24 | 40 |

[a] t = 0 is the time taken when DMSO was added.; [b] t = 0 is the time taken when $D_2O$ was added.

In the case of complexes **8** and **13**, rapid Cp hydrolysis occurred within 15 min (Figure 1b,1c), and after 24 h the singlets due to the metal bound Cp rings had completely disappeared and only the characteristic cyclopentadiene (CpH) multiplets were observed. Solutions of **8** also darkened after 24 h. As in the case of **1**, a downfield DMSO multiplets appeared in these experiments and were assigned tentatively to DMSO coordinated to titanium. This DMSO-Ti signal increased in intensity with time and was the predominant species present after 24 h. A new resonance also appeared in the spectra at around 8.4 ppm (Figure 1b, 1c and 1d) and was tentatively assigned as a binuclear degradation species similar to those reported previously, (eg., $[Cp_2Ti(X)O(X)TiCp_2]^{2+}$ [18]). This resonance was sharp in solutions of complex **8** and broad in solutions where glycine was present (Figure 1c and 1d).

In order to establish whether glycine, which appears to rapidly dissociate from the metal when **13** is dissolved in DMSO, promotes the Cp hydrolysis reaction, glycine ($HOOCCH_2NH_2$, 2 mol equivalents) was added to a solution of $Cp_2TiCl_2$ **1** and NMR spectra were recorded over 24 h. After 15 min, approx 40% Cp hydrolysis had occurred, while after 3 h significant Cp hydrolysis (80%) was observed (Figure1d, Table 3). Thus, free glycine does promote the Cp hydrolysis reaction, but the rate is slower than in the case of bisglycine derivative **13** in which the glycine ligands are initially coordinated to the metal centre in the zwitterioninc form.

***Hydrolysis in 10%DMSO/90%$D_2O$***

Two different preparation methods were used (i) the sample was dissolved directly in 10%DMSO/$D_2O$ solution and (ii) the sample was dissolved in DMSO and then diluted with $D_2O$ (i.e., the method used for sample administration [19]) with accurate recording of the time the sample was allowed to stand in DMSO before dilution with water.

(i) The rate of Cp hydrolysis of the complexes $Cp_2TiX_2$ (X = Cl **1**, $OOCCCl_2$ **8**, glycine **13**; 10 μmol) directly dissolved in 10%DMSO/90%$D_2O$ solutions is summarised in Table 3. The Cp hydrolysis was estimated at <2% over 24 h for **1** and **8** as the amount of signal from CpH in the NMR spectra was negligible. In the case of **13** the amount of bound Cp signal was reduced by 20% compared to the $CH_2$ signal of glycine after 24 h and was attributed to Cp hydrolysis which resulted in precipitation of a Cp containing species. For all complexes, the amount of precipitate was negligible within 3 h and 20% for **13** after 24 h. The results confirm that 10%DMSO/90%$D_2O$ does not have a significant effect on Cp hydrolysis over several hours when this preparation method is employed, and the results are very similar to those obtained in water.

(ii) Table 4 summarises the results obtained for the complexes $Cp_2TiX_2$ (X = Cl **1**, $OOCCCl_3$ **8**, glycine **13**; 10 μmol) when they were initially dissolved in 50 μl DMSO and left to stand for 0.5, 3 and 10 min before addition of 450 μl of $D_2O$. In the case of titanocene dichloride **1** minimal precipitate was detected with time and there was no evidence of free Cp in solution or the presence of any dicyclopentadiene (spectra not shown).

In the case of titanocene bis(glycine) **13** precipitation was observed to increase with time. The % Cp hydrolysis was estimated by comparing the integral areas for the bound Cp resonance to that for the $CH_2$ resonance of the glycine ligand (spectra not shown). The results show that the % Cp hydrolysis increased when the length of time that titanocene bis(glycine) **13** was allowed to stand in DMSO was increased (Table 4). Further Cp hydrolysis was almost completely inhibited upon dilution with $D_2O$.

Hydrolysis with titanocene bis(trichloroacetate) **8** was evident from both the formation of precipitate with time and darkening of solutions. The initial concentration of complex dissolved in DMSO was also important; lowering the concentration of the complex (10 μmol to 6 μmol) followed by dilution with $D_2O$ showed only a very weak Cp signal in the NMR spectrum, and Cp hydrolysis was estimated at > 90 % with the lower concentration of complex

### DISCUSSION

While the antitumour activity of titanocene dichloride **1** has been recognised since 1980, and the drug has been extensively tested against a wide range of tumours [1-3], the chemical basis for the mechanism of antitumour action is poorly understood. Due to the limited aqueous solubility of $Cp_2TiCl_2$ **1**, and the formation of precipitates at high pH, titanocene dichloride **1** has been administered in 10%DMSO/90%saline solutions at low pH. The complex is typically dissolved in DMSO and then diluted to the required volume [19]. In order to overcome potential problems associated with precipitation upon intravenous administration and increase the bioavailability of the drug, a number of active derivatives with improved aqueous solubility have been reported [11,12]. In the case of complexes **7**, **8** and **9**, the introduction of hydrophilic ligands resulted in (i) diminution of toxic properties (ii) widening of the therapeutic range and (iii) an increase in aqueous solubility for **7** and **9** which allowed the complexes to be administered in pure saline [11]. Moreover, a further reduction in toxic properties was attained by elevation of the pH of the injected solution of complex **7** in a manner similar to titanocene dichloride **1** [19]. However, in contrast to other carboxylated complexes, the trichloroacetate derivative **8** did not exhibit improved aqueous solubility, and hence administration was only carried out in 10%DMSO [11]. The bisglycine derivate **13**, while exhibiting excellent aqueous solubility, was tested in 10%DMSO, presumably to allow comparison of the results with all previous studies [13].

Due to the use of DMSO in therapeutic mixtures, we have examined the relative stabilities of metallocenes **1**, **8** and **13** in three different solutions. Complex **1** was studied as the parent metallocene undergoing clinical trials. The bisglycine analogue **13** was studied due to the unexpected, reduced antitumour activity of this complex [13], and complex **8** was selected as a representative of the hydrophilic carboxylate series [11] which has been screened against a number of tumour types [20]. Comparison of the rate of Cp hydrolysis of these three metallocenes in water, DMSO and 10%DMSO/90%$D_2O$ show several distinct trends. Firstly, the rate of Cp hydrolysis of derivatives **8** and **13** is accelerated in DMSO compared with the parent compound **1**. Secondly, the hydrolysis reaction(s) in DMSO [15] to inactive titanocene species are highly concentration dependent, as diluting the DMSO to ~10% with water effectively prevents further degradation from occurring. Thus, for samples prepared in 10%DMSO, the amount of time between when the sample is dissolved and then diluted with water is significant; after several minutes as much as 40% of the species present in DMSO no longer contains the "$Cp_2Ti$" moiety in which the Cp rings remain coordinated to the titanium metal centre.

The rate of halide and Cp ring hydrolysis in titanocene dichloride **1** in water has been well-characterised [6]. Rapid hydrolysis of the two halide ligands occurs in a series of equilibrium reactions to give a solution of pH ~ 1 in which the Cp rings remain metal bound for > 24 h. However, at higher pH values protonolysis of the Cp rings occurs to give cyclopentadiene and dicyclopentadiene as well as insoluble, uncharacterised hydrolysis products. The exact nature of the pseudohalide ligands have not been identified with $Cp_2TiCl_{2(aq)}$ likely to contain a number of species such as $Cp_2Ti(H_2O)Cl^+$, or $Cp_2Ti(H_2O)_x(OH)_y{}^{(2-y)+}$. If we consider the effect of DMSO on this equilibrium, then our results (Figure 1, Table 3) show that the rate of Cp hydrolysis in DMSO is slightly faster than in pure water at low pH. Direct evidence for formation of one or more species in which DMSO coordinates to the metal centre is provided by the appearance of new DMSO multiplets in the $^1H$ NMR spectrum. However, more complete characterisation of these degradation products was not possible.

In the case of **8** and **13**, in the presence DMSO the rate of Cp hydrolysis is dramatically increased compared to aqueous solutions of these complexes in water at low pH. While the exact reasons for this and the mechanism by which the hydrolysis/displacement reactions occur are not fully understood, there are a number of factors that may contribute to the results. Firstly, the lability of the carboxylate-Ti bond in DMSO (and the liberated conjugate base of the carboxylic acid), appear to be important in both **8** and **13** as the chloride ligands in titanocene dichloride **1** behave differently. In the case of $Cp_2TiCl_2$ **1**, the chloride ligands dissociate and generate the coordinating subunit "$Cp_2Ti^{2+}$" only when dissolved in water, while in DMSO, dissociation of the Cl-Ti bond is much slower. In contrast, in the case of **8** and **13**, in DMSO both the carboxylate and Cp hydrolysis reactions are fast. While DMSO contains a minor amount of water, the major role of the DMSO solvent in the hydrolysis chemistry appears to be to displace the halide/carboxylate ligand and activate the Cp ring to protonolysis.

As the major focus of our studies on antitumour metallocenes has been to attempt to correlate the chemical stability and coordination chemistry of these complexes with their observed antitumour properties, and thus provide the basis for rational drug design, we now compare the hydrolysis results with the antitumour data in the literature. In the case of the glycine derivative **13**, our results suggest that administration of the complex in pure water should give improved results compared to those in 10%DMSO. As the time between dissolving the complex in DMSO and dilution with water is not given in the Experimental [13] (and is generally not important), it is impossible to state unequivocally that Cp hydrolysis is responsible for very surprising reduced activity results (Table 1). However, partial Cp hydrolysis in DMSO does offer a simple explanation for the results and still supports the general hypothesis that the biologically active species generated *in vivo* is "$Cp_2Ti^{2+}$". A unique feature of the amino acid complexes **13** and **14**, which may play a role in the biological activity, is that they are zwitterionic and are present as the hydrochloride salt in the complex. However, the thiophenol derivative **7** was also tested as the hydrochloride salt and found to have maximum activity against EAT tumours (Table 1). It is interesting that only sporadic cure rates, and the absence of strong dose-activity relationships was observed for the corresponding orthoderivative **17** (Table 2) in which the amine is present in neutral form, hinting that these sidechains may affect the subsequent chemistry.

The antitumour results obtained with the trichloroacetate derivative **8** are more difficult to rationalise on a chemical basis (Table 1). This derivative retains the activity of the parent derivative but the dose required to achieve an optimal cure rate is significantly increased compared to other titanocene derivatives. Partial decomposition in DMSO could contribute to this dosage but further comparative tests on a range of related titanocenes would be required to confirm this hypothesis.

### CONCLUSIONS

Our hydrolysis studies suggest that titanocene derivatives in which X = Cl, Br, I retain maximum antitumour activity. Certain carboxylate ligands such as in the complexes **8** and **13** promote hydrolysis/degradation reactions to biologically inactive species in DMSO in a process that is DMSO concentration dependent. These results are consistent with the reduced activity observed for the bisglycine analogue **13** and suggest that administration in the absence of DMSO (as the complex is highly water soluble) or direct dissolution in 10%DMSO/90%saline may result in modified cure rates. Direct dissolution of complex **8** in 10%DMSO/90%saline may also result in a modified cure rate and optimum dose. The results emphasise the importance of examination of the effect of coordinating solvents on the stability of metallocene complexes.

In terms of medicinal chemistry and the design and new transition metal drugs based on the titanocene framework, our results support antitumour studies that indicate that a range of X substituents may be incorporated into the $Cp_2TiX_2$ structure without loss of activity. However, certain carboxylate based ligands appear to have reduced stability in DMSO solutions. Hence the chemical hydrolysis reactions that occur in both water at low and elevated pH as well as the effect of DMSO on new titanocene based anticancer drugs, needs to be taken into account in derivatives that contain acid functional groups in the X ligands.

**ACKNOWLEDGMENTS**
The award of an Australian Postgraduate Award (G.M.) is gratefully acknowledged.

### REFERENCES

[1] Köpf-Maier, P.; Köpf, H. *Drugs of the Future* **1986**, *11*, 297
[2] Köpf-Maier, P.; Köpf, H. *Struct. Bond.* **1988**, *70*, 105
[3] Köpf-Maier, P.; Köpf, H. in *Metal Compounds in Cancer Chemotherapy* Ed. Fricker, S. P.; Chapman & Hall, London, **1994**, 109.
[4] Berdel, W. E.; Schmoll, H. J.; Scheulen, M. E.; Korfel, A.; Knoche, M. F.; Harstrci, A.; Bach, F.; Baumgart, J.; Sass, G. *J. Cancer Res. Clin. Oncol.* **1994**, *120[Suppl]*, R172; Moebus, V. J.; Stein, R.; Kieback, D. G.; Runnebaum, I. B.; Sass, G.; Kreienberg, R. *Anticancer Research* **1997**, *17*, 815.
[5] The term "$Cp_2Ti_2^{+}$" is used in the text to refer to the predominant coordinating species present when $Cp_2TiCl_2$ is dissolved in water. Solutions of $Cp_2TiCl_2$ are likely to contain a number of species such as $Cp_2Ti(H_2O)Cl^+$, or $Cp_2Ti(H_2O)_x(OH)_y^{(2-y)+}$
[6] Toney, J. H.; Marks, T. J. *J. Am. Chem. Soc.* **1985**, *107*, 947.
[7] Köpf-Maier, P.; Köpf, H. *J. Organomet. Chem.* **1988**, *342*, 167.
[8] Köpf-Maier, P.; Grabowski, S.; Köpf, H. *Eur. J. Med. Chem.* **1984**, *19*, 347.
[9] Köpf-Maier, P.; Kahl, W.; Klouras, N.; Hermann, G.; Köpf, H. *Eur. J. Med. Chem.* **1981**, *16*, 275.
[10] Köpf-Maier, P.; Klapötke, T.; Köpf, H. *Inorg. Chim. Acta* **1988**, *153*, 119.
[11] Köpf-Maier, P; Grabowski, S.; Liegener, J.; Köpf, H. *Inorg. Chim. Acta* **1985**, *108*, 99.
[12] Klapötke, T. M.; Köpf, H.; Tornieporth-Oetting, I. C.; White P. S. *Organometallics* **1994**, *13*, 3628; Klapötke, T. M.; Köpf, H.; Tornieporth-Oetting, I. C.; White P. S. *Angew. Chem. Int. Ed. Engl.* **1994**, *33*, 1518.
[13] Köpf-Maier, P.; Tornieporth-Oetting, I. C. *Biometals.* **1996**, *9*, 267.
[14] Mokdsi, G.; Harding, M.M. *J. Organomet. Chem.* **1998**, in press.
[15] The term Cp hydrolysis is used to describe reactions in which the Cp ligands are replaced by, or react with, water and/or DMSO. Hydrolysis (or substitution) of the halide ligands by water and/or DMSO can also occur. These reactions give rise to both soluble hydrolysis products and insoluble precipitates. In 100% DMSO, the mechanism by which the Cp and halide ligands are replaced is not by reaction with water (ie hydrolysis), except for possibly the minor amount of water that is also present in DMSO. For comparative purposes under the three solvent systems studied, the term hydrolysis is used throughout the text.
[16] Glasoe, P. K.; Long, F. A. *J. Phys. Chem.* **1960**, *64*, 188.
[17] Murray, J.H.; Harding, M.M. *J. Med. Chem.* **1994**, *37*, 1936.
[18] Döppert, K. *Makromol. Chem. Rapid Comm.* **1980**, *1*, 519.
[19] Köpf-Maier, P; Hesse, B.; Voigtlander, R.; Köpf, H. *J. Cancer Res Clin Oncol.* **1980**, *97*, 31.
[20] Köpf-Maier, P.; Klapötke, T.; Köpf, H.; Preiss, F.; Marx, T. *Anticancer Res.* **1986**, *6*, 33.

**Received: July 1, 1998 - Accepted: July 14, 1998**

